# Supplementary material for: Inflammation and autoimmunity are interrelated in patients with sickle cell disease at a steady-state condition: implications for vaso-occlusive crisis, pain, and sensory sensitivity
Source: Front Immunol. 2024 Feb 1;15:1288187. doi: 10.3389/fimmu.2024.1288187 (PMC10867278; doi:10.3389/fimmu.2024.1288187)
Supplement: Supplementary file 1 [file Table_1.pdf]

Supplementary Table 1: Comparison of plasma levels of the 80 mediators in patients with SCD and HC

**Soluble factors**	**HC (n=18)**	**SCD (n=36)**	***p* value**	**Adjusted p value**
APRIL	470 (262.8-1708)	512.7 (106.4-960.1)	0.51531	0.99999
BAFF	3.053 (1.051-4.074)	4.283 (2.555-6.08)	**0.01758**	0.62295
BLC	37.39 (24.86-63.05)	29.79 (21.82-60.22)	0.70633	0.99999
bNGF	1.138 (0.875-3.056)	2.05 (1.186-4.613)	**0.02762**	0.75348
CCL1	0.68 (0.525-1.16)	1.163 (0.515-2.688)	0.09610	0.98243
CCL17	2.12 (1.199-5.145)	2.093 (1.551-4.935)	0.49238	0.99999
CCL21	0.075 (0-12.78)	20.41 (0.585-71.48)	**0.00280**	0.17337
**CCL23**[&]	486.5 (302-639.3)	873.8 (574.4-1285)	**0.00014**	**0.01046**
CCL25	54.98 (37.58-94.92)	37.44 (25.94-98.24)	0.14983	0.99710
CD30	76.7 (59.84-94.06)	77.14 (52.44-174.8)	0.73350	0.99999
CD40-Ligand	0 (0-26.31)	2.16 (0-47.46)	0.34574	0.99998
CXCL6	10.88 (6.814-20.09)	6.323 (5.009-17.41)	0.12176	0.99368
ENA-78	75.46 (28.35-146)	77.33 (33.99-149.7)	0.72300	0.99999
Eotaxin	8.845 (5.774-16.38)	7.713 (4.1-13.23)	0.42019	0.99998
Eotaxin-2	0.13 (0-6.589)	10.4 (0-97.67)	**0.04479**	0.88393
Eotaxin-3	0.9275 (0.6513-1.599)	1.075 (0.8788-1.685)	0.22639	0.99955
FGF-2	0 (0-2.36)	1.043 (0-10.04)	0.06400	0.94181
Fractalkine	4.96 (2.651-8.59)	3.785 (1.334-8.701)	0.49209	0.99999
**Gal-3**[&]	6749 (4949-8982)	15144 (6910-34355)	**0.00055**	**0.03958**
G-CSF/CSF-3	4.213 (0-89.43)	50.68 (25.34-116.3)	**0.01299**	0.51903
GM-CSF	0 (0-0)	3.785 (0-20.54)	**0.00256**	0.16236
Granzyme A	3.27 (0-10.95)	11.12 (4.63-23.48)	**0.00442**	0.25324
Granzyme B	10.43 (7.018-28.02)	20.19 (12.57-31.93)	**0.01758**	0.62295
GRO-α	1.745 (1.339-2.039)	2.443 (1.745-3.144)	**0.00625**	0.33050
**HGF**[&]	6.425 (2.965-20.29)	56.21 (13.12-70.23)	**0.00003**	**0.00253**
IFN-α	1.33 (0.5938-2.626)	1.283 (0.6538-3.098)	0.55132	0.99999
**IFN-γ**[&]	1.81 (1.348-2.235)	3.89 (2.303-4.81)	**0.00023**	**0.01737**
IL-1α	3.908 (2.531-11.02)	6.933 (4.131-16.35)	**0.04832**	0.89752
IL-1β	0 (0-0.035)	0 (0-1.826)	0.13532	0.99539
IL-2	4.253 (3.161-6.196)	9.093 (4.505-14.86)	**0.00940**	0.43242
IL-2R	1565 (1181-2125)	1845 (968.3-2445)	0.66921	0.99999
IL-3	11.6 (1.09-71.92)	19.94 (1.09-75.18)	0.77365	0.99999
IL-4	19.38 (11.17-35.13)	37.34 (18.45-77.21)	**0.00901**	0.42436
IL-5	0 (0-0.2225)	3.49 (0-12.34)	**0.00290**	0.17675
IL-6	0 (0-0.7625)	1.49 (0-7.418)	**0.03843**	0.84757
IL-7	0 (0-0)	0 (0-1.123)	**0.02305**	0.70251
IL-8	0.745 (0.075-6.974)	5.545 (2.09-10.21)	**0.01183**	0.49255
IL-9	0.225 (0-1.443)	1.42 (0.4813-3.026)	**0.01134**	0.48383
IL-10	0 (0-0)	0 (0-0)	0.21105	0.99936
IL-12p70	0 (0-0)	0 (0-0)	>0.999999	>0.999999
IL-13	0 (0-0)	0 (0-0)	0.15442	0.99718
IL-15	3.023 (1.763-5.186)	4.52 (2.743-7.299)	0.08540	0.97647
IL-16	112.9 (88.25-139.9)	110.6 (72.38-181.9)	0.99275	0.99999
IL-17A	0 (0-1.725)	0.98 (0-3.959)	0.05482	0.91633
**IL-18**[&]	5.025 (3.444-7.061)	13.73 (7.218-50.83)	**0.00002**	**0.00173**
IL-20	6.853 (5.543-10.49)	9.553 (6.715-21.82)	**0.02509**	0.72631

IL-21	19.8 (9.194-42.43)	18.97 (11.98-43.21)	0.76780	0.99999
IL-22	0 (0-4.548)	0.115 (0-5.458)	0.36868	0.99998
IL-23	0 (0-9.943)	0.995 (0-19.3)	0.19284	0.99915
IL-27	0 (0-0)	0 (0-9.915)	0.12970	0.99490
IL-31	3.585 (0-14.49)	7.28 (0-21.49)	0.34373	0.99998
**IL-34**[&]	11.52 (8.323-18.46)	21.92 (13.71-43.89)	**0.00051**	**0.03716**
IL-37	0 (0-0)	0 (0-0)	0.35250	0.99998
**IP-10**[&]	17.22 (10.13-25.58)	36.93 (21.25-60.65)	**0.00012**	**0.00891**
I-TAC	18.83 (7.979-38.96)	17.72 (8.79-43.49)	0.57908	0.99999
LIF	0.59 (0.375-2.696)	2.435 (1.118-4.755)	**0.00991**	0.44448
MCP-1	33.5 (17.6-50.59)	22.9 (14.02-41.95)	0.19778	0.99915
**MCP-2**[&]	0.1725 (0.07375-0.6713)	2.138 (0.6813-5.304)	**0.00001**	**0.00091**
MCP-3	0 (0-4.415)	0.51 (0-9.279)	0.40791	0.99998
MCP-4	7.753 (3.664-19.27)	12.26 (4.586-20.97)	0.38924	0.99998
M-CSF	0.16 (0-8.69)	2.505 (0.3675-7.083)	0.15583	0.99718
MDC/CCL22	55.35 (27.21-64.36)	71.08 (40.77-125.2)	0.08977	0.97885
MIF	73.91 (49.99-81.74)	75.76 (43.81-119.4)	0.57908	0.99999
MIG	15.04 (12.3-30.81)	20.53 (12.76-36.63)	0.48353	0.99999
MIP-1α	0 (0-1.558)	2.73 (0.6875-6.959)	**0.00629**	0.33050
MIP-1β	30 (14.85-40.48)	56.77 (29.67-71.46)	**0.00134**	0.09067
MIP-2α	2.543 (1.43-4.888)	3.405 (1.385-8.048)	0.38695	0.99998
MIP-β	150.7 (76.14-199.7)	172.8 (106-252.2)	0.31165	0.99996
MIP-3α	5.12 (4.388-5.503)	5.97 (5.46-7.559)	**0.00196**	0.12821
MMP-1	105 (63.18-178.2)	176 (5.97-267.2)	0.30101	0.99996
**PTX3**[&]	1137 (797.8-1924)	3045 (1557-5283)	**0.00064**	**0.04533**
SCF	4.6 (3.17-7.959)	4.115 (1.706-7.603)	0.28394	0.99994
TNF-alpha	2.155 (1.976-2.63)	2.68 (2.31-3.211)	**0.00460**	0.25884
TNF-beta	0.0725 (0-2.181)	1.46 (0.44-4.823)	**0.02021**	0.66116
TNF-RII	92.57 (84.13-118.5)	88.11 (56.77-136.1)	0.46102	0.99999
TRAIL	13.09 (4.828-53.93)	10.05 (2.005-36.4)	0.65920	0.99999
TREM-1	0 (0-235.4)	248.2 (16.25-761.1)	**0.02832**	0.75524
TSLP	1.328 (0.7763-2.208)	2.323 (1.663-3.431)	**0.00791**	0.38881
Tweak	1241 (937.1-1604)	988.6 (822.1-1870)	0.51835	0.99999
VEGF-A	50.02 (33.59-65.04)	73.17 (35.73-156.1)	0.05228	0.91076

Note: Plasma levels of the 80 analytes (pg/ml) are shown as median and interquartile range. The Mann-Whitney test was used to compare differences between patients with SCD and healthy controls (HC) without and with Holm-Šídák correction for multiple comparisons. $p < 0.05$ was considered significant and shown in bold., [&]mediators with adjusted p < 0.05 in Table 3 and shown in bold.
