# Supplementary material for: Inflammation and autoimmunity are interrelated in patients with sickle cell disease at a steady-state condition: implications for vaso-occlusive crisis, pain, and sensory sensitivity
Source: Front Immunol. 2024 Feb 1;15:1288187. doi: 10.3389/fimmu.2024.1288187 (PMC10867278; doi:10.3389/fimmu.2024.1288187)
Supplement: Supplementary file 2 [file Table_2.pdf]

Supplementary Table 2: Fold change of median concentration of the 37 factors elevated in patients with SCD relative to healthy controls

Soluble factors	Fold change	Soluble factors	Fold change
MIP-1α	NA	**Gal-3[&]**	2.24
GM-CSF	NA	IFN-γ	2.15
IL-7	NA	IP-10	2.14
IL-5	NA	IL-2	2.14
IL-6	NA	Granzyme B	1.94
IL-17A	NA	IL-4	1.93
TREM-1	NA	**IL-34[&]**	1.90
CCL21	272.13	MIP-1β	1.89
Eotaxin-2	80.00	bNGF	1.80
TNF-β	20.14	**CCL23[&]**	1.80
**MCP-2[&]**	12.39	IL-1α	1.77
G-CSF/CSF-3	12.03	TSLP	1.75
**HGF[&]**	8.75	VEGF-A	1.46
IL-8	7.44	BAFF	1.40
IL-9	6.31	GRO-α	1.40
LIF	4.13	IL-20	1.39
Granzyme A	3.40	TNF-α	1.24
**IL-18[&]**	2.73	MIP-3α	1.17
**PTX3[&]**	2.68		

Note: NA, not applicable due to the median value of 0 in healthy controls, [&]mediators with adjusted $p < 0.05$ in Table 3 and shown in bold.
